# Severe canine distemper outbreak in unvaccinated dogs in Mozambique

**DOI:** 10.4102/jsava.v87i1.1350

**Published:** 2016-07-15

**Authors:** Julieta Zacarias, Alberto Dimande, Sara Achá, Paula T. Dias, Elisa M. Leonel, Aurora Messa, Baltazar Macucule, José L. Júnior, Custódio G. Bila

**Affiliations:** 1 Mozambican Institute of Agriculture Research, Maputo, Mozambique; 2 Faculty of Veterinary Medicine, Eduardo Mondlane University, Mozambique; 3 Ministry of Agriculture and Food Security, Maputo, Mozambique

## Abstract

Although significant animal suffering caused by preventable diseases is frequently seen in developing countries, reports of this are scarce. This report describes avoidable animal suffering owing to a suspected canine distemper (CD) outbreak in unvaccinated dogs owned by low-income families in Mozambique that killed approximately 200 animals. Affected dogs exhibited clinical signs, and gross and microscopic lesions compatible with CD. Immunohistochemical staining confirmed the presence of canine distemper virus (CDV) in the kidney of one dog from the cohort. This brief communication again illustrates that large outbreaks of CDV in unvaccinated dogs occur and that large-scale avoidable suffering and threats to the health of dogs and wild canines continue. Mass vaccination supported by government and non-government organisations is recommended.

## Introduction

Canine distemper (CD) is a highly contagious, acute or subacute systemic viral disease that has a high mortality rate in dogs and wild canines. It is caused by canine distemper virus (CDV), which belongs to the genus *Morbillivirus* within the subfamily *Paramyxovirinae*.

Vaccination remains the principal strategy for protection, and once clinical signs develop, treatment is limited to supportive care. Even in settings where a diagnosis is rapidly reached, a high standard of care is available and high levels of vaccination are achieved, CD outbreaks continue to occur amongst domestic dogs (Bohm, Blixenkrone & Lund 1989; Ek-Kommonen *et al*. 1997; Ezeibe 2005; Gard *et al*. 2013; Gemma *et al*. 1996; Maes *et al*. 2003; Martella, Elia & Buonavoglia 2008; Patronek *et al*. 1995; Schumaker *et al*. 2012; Willi *et al*. 2015).

This report pertains to a severe suspected CDV outbreak in unvaccinated domestic dogs in Mozambique, owned mainly by low-income families, who receive no care from state or private veterinarians. This observational report is, to our knowledge, the only documentation of a CDV outbreak of this magnitude in Africa in this species.

## Case presentation

Cases of sick dogs mainly from low-income families were detected by State Veterinarians in Nampula City, in the northern region of Mozambique, during January and February of 2013. About 200 dogs of all ages died. Affected dogs had never been vaccinated against CDV and consisted of different breeds, with predominance of the Africanis breed. State veterinarians clinically examined 25 cases and described the following symptoms: anorexia, fever, weakness, nasal and ocular mucopurulent discharges, dyspnoea, coughing, pale mucous membranes, muscular trembling and/or clonus, diarrhoea, resulting in fatality within 7–14 days. Treatment with antibiotics was unsuccessful.

Three cases were submitted for post-mortem examination and samples with relevant lesions were collected for histopathology and immunohistochemistry. Post-mortem examinations were carried out following standard procedures. Tissue samples were preserved in 10% phosphate-buffered formaldehyde, embedded in paraffin wax, sectioned at 5 μm thickness and stained with haematoxylin-eosin for routine histopathological examination. Sections of brain, lung, heart, kidney and urinary bladder were immunolabelled for CDV antigen with a monoclonal mouse antibody against CDV (Agriculture Canada, Animal Diseases Research, Canada).

The most obvious gross post-mortal lesions were cachexia (3/3), mucopurulent exudate in the trachea and bronchi (2/3), focal pneumonia (3/3), cyanotic heart (2/3), splenic atrophy with increased consistency (2/3), pale kidneys (3/3) and contracted bladders with multifocal haemorrhages in the mucosa (2/3).

Histopathological examination of the lung, bronchi and bronchioli showed extensive areas of severe purulent inflammation (2/3). Splenic and lymph node tissues appeared moderately active to highly active (2/3). In the grey matter of the cerebellum, multifocal blood vessels showed notable leukostasis, predominantly with lymphocytes and a few macrophages, and there were also mononuclear infiltrates visible in the perivascular spaces in these areas. There was mild gliosis in the tissue. A few blood vessels in the meninges also showed perivascular cuffing with mononuclear leukocytes, predominately lymphocytes (2/3). A few multifocal aggregates of neutrophils were visible in the parenchyma of the liver, and a mild degree of leukostasis was evident in the sinusoids. The portal regions showed the presence of leukocytes (3/3). Focally extensive severe interstitial inflammation of a chronic lymphoplasmocytic appearance was seen in the prostate (3/3).

The history of the disease, the clinical signs and gross and histopathological presentations raised the probability of the presence of a CDV infection. To support this putative diagnosis, immunohistochemistry was performed, which confirmed the disease through positive immunoreactivity to CDV antigens in one of these three cases.

The incidence of CDV infections worldwide has decreased thanks to the introduction of the highly protective CDV-modified live virus (MLV) vaccines more than 60 years ago (Martella *et al*. 2008). However, in regions with a low proportion of vaccinated dogs, the incidence of CDV epidemics is still high (Willi *et al*. 2015). Vaccination against CDV in Mozambique is only available to people who can afford private veterinary care and therefore the herd immunity against this disease falls far short of the 80% required to prevent outbreaks (Horzinek 2006).

This report illustrates the highly contagious nature of the disease and the threat to animal welfare posed by non-vaccination. Valuable wildlife would also potentially be threatened by this disease in dogs.
